# Seroprevalence, Risk Factors and Maternal–Fetal Outcomes of *Toxoplasma gondii* in Pregnant Women from WHO Eastern Mediterranean Region: Systematic Review and Meta-Analysis

**DOI:** 10.3390/pathogens12091157

**Published:** 2023-09-12

**Authors:** Ali A. Rabaan, Leonard Ighodalo Uzairue, Amal H. Alfaraj, Muhammad A. Halwani, Muzaheed, Abdulsalam Alawfi, Amer Alshengeti, Nawal A. Al Kaabi, Eman Alawad, Mashael Alhajri, Sara Alwarthan, Abeer N. Alshukairi, Souad A. Almuthree, Roua A. Alsubki, Nada N. Alshehri, Mohammed Alissa, Hawra Albayat, Tasneem I. Zaidan, Hassan Alagoul, Ali Al Fraij, Jeehan H. Alestad

**Affiliations:** 1 Molecular Diagnostic Laboratory, Johns Hopkins Aramco Healthcare, Dhahran 31311, Saudi Arabia; 2 College of Medicine, Alfaisal University, Riyadh 11533, Saudi Arabia; 3 Department of Public Health and Nutrition, The University of Haripur, Haripur 22610, Pakistan; 4 Department of Medical Laboratory Science, Federal University, Oye-Ekiti 371104, Ekiti State, Nigeria; 5 Pediatric Department, Abqaiq General Hospital, First Eastern Health Cluster, Abqaiq 33261, Saudi Arabia; 6 Department of Medical Microbiology, Faculty of Medicine, Al Baha University, Al Baha 47810, Saudi Arabia; 7 Department of Clinical Laboratory Science, College of Applied Medical Sciences, Imam Abdulrahman Bin Faisal University, Dammam 31441, Saudi Arabia; 8 Department of Pediatrics, College of Medicine, Taibah University, Al-Madinah 41491, Saudi Arabia; 9 Department of Infection Prevention and Control, National Guard Health Affairs, Prince Mohammad Bin Abdulaziz Hospital, Al-Madinah 41491, Saudi Arabia; 10 Department of Pediatric Infectious Disease, Sheikh Khalifa Medical City, Abu Dhabi 51900, United Arab Emirates; 11 College of Medicine and Health Science, Khalifa University, Abu Dhabi 127788, United Arab Emirates; 12 Adult Infectious Diseases Department, Prince Mohammed Bin Abdulaziz Hospital, Riyadh 11474, Saudi Arabia; 13 Department of Internal Medicine, College of Medicine, Imam Abdulrahman Bin Faisal University, Dammam 34212, Saudi Arabia; 14 Department of Medicine, King Faisal Specialist Hospital and Research Center, Jeddah 21499, Saudi Arabia; 15 Department of Infectious Disease, King Abdullah Medical City, Makkah 43442, Saudi Arabia; 16 Department of Clinical Laboratory Sciences, College of Applied Medical Sciences, King Saud University, Riyadh 11362, Saudi Arabia; 17 Internal Medicine Department, College of Medicine, King Khalid University Medical City, Abha 61481, Saudi Arabia; 18 Department of Medical Laboratory Sciences, College of Applied Medical Sciences, Prince Sattam Bin Abdulaziz University, Al-Kharj 11942, Saudi Arabia; 19 Infectious Disease Department, King Saud Medical City, Riyadh 7790, Saudi Arabia; 20 Pediatric Department, King Abdulaziz Hospital, Jeddah 23831, Saudi Arabia; 21 Blood Bank Section, Dammam Regional Laboratory and Blood Bank, Dammam 31411, Saudi Arabia; 22 Medical Laboratories & Blood Bank Department, Jubail Health Network, Eastern Health Cluster, Ministry of Health, Jubail 35514, Saudi Arabia; 23 Immunology and Infectious Microbiology Department, University of Glasgow, Glasgow G1 1XQ, UK; 24 Microbiology Department, Collage of Medicine, Jabriya 46300, Kuwait

**Keywords:** *Toxoplasma gondii*, pregnancy, Eastern Mediterranean, stillbirth, miscarriage

## Abstract

Background: The protozoan parasite *Toxoplasma gondii* may cause serious illness in the immunocompromised. The *Toxoplasma gondii* seropositive prevalence in pregnant women in WHO Eastern Mediterranean Region countries is inconsistent in the literature and it is associated with outcomes that have not be fully elucidated, hence the need for a better understanding of the pooled seroprevalence and associated maternal and fetal outcomes. Objective: The objective was to conduct a systematic literature review and determine the pooled prevalence of WHO Eastern Mediterranean Regional countries’ pregnant women’s seroprevalence of *Toxoplasma gondii* and the maternal–fetal outcomes. Methods: This quantitative study examined WHO Eastern Mediterranean countries’ maternal–fetal outcomes and *Toxoplasma gondii* prevalence in pregnant women. The targeted population was pregnant women, while the primary outcome was seropositivity of *Toxoplasma gondii*, while other outcomes such as maternal and fetal associations and risk factors were determined PubMed, SCOPUS, MEDLINE, and Index Medicus for the Eastern Mediterranean Region (IMEMR) databases were searched up until 30 January 2023. The search terms used were “*Toxoplasma gondii*” OR “*Toxoplasma* infection” AND “Pregnant woman” or pregnan* OR Antenatal OR Prenatal OR Gravidity OR Parturition OR Maternal AND WHO Eastern Mediterranean Region). OpenMeta-Analyst and Jamovi were used to analyze the generated data. Results: In total, 95 of 2947 articles meeting the inclusion criteria examined *Toxoplasma gondii* prevalence in pregnant women from WHO Eastern Mediterranean countries. The pooled prevalence of *Toxoplasma gondii* in pregnant women was 36.5% (95%CI: 32.6–40.4) with a median value of 35.64%, range values of 1.38–75.30%, with 99.61% heterogeneity. The pooled seroprevalence of IgG of *Toxoplasma gondii* was 33.5% (95%CI: 29.8–37.2) with a median value of 33.51%, and a range values of 1.38–69.92%; the pooled seroprevalence of IgM was 3.6% (95%CI: 3.1–4.1)) with a median value of 3.62 and range values of 0.20–17.47%, while cases of pooled seroprevalence of both IgG and IgM positivity was 3.0% (95%CI: 1.9–4.4) with a median value of 2.05 and a range values of 0.05–16.62%. Of the *Toxoplasma gondii* seropositive women, 1281/3389 (34.8%) 174/1765 (32.9%), 1311/3101 (43.7%), and 715/1683 (40.8%) of them had contact with cats, drank unprocessed milk, ate raw or undercooked meat and ate unwashed raw vegetables, respectively. The maternal–fetal outcomes associated with *Toxoplasma gondii* seropositivity were a history of abortions, miscarriage, stillbirth, intrauterine fetal death, and premature birth, which were found in 868/2990 (32.5%), 112/300 (36.1%), 111/375 (25.7%), 3/157 (1.9%) and 96/362 (20.1%) of women who tested positive for *Toxoplasma gondii* antibodies. Conclusion: The study found a high proportion of *Toxoplasma gondii* seroprevalence in pregnant women in the WHO Eastern Mediterranean Region, which may be linked to poor outcomes for mothers and their babies. Thus, pregnant women require monitoring and comprehensive prevention strategies for *Toxoplasma gondii* infection.

## 1. Introduction

*Toxoplasma gondii* is one of the most common parasites that affect humans. It can result in toxoplasmosis [1,2,3]. While healthy people may have mild infections, pregnant women, unborn infants, and others with impaired immune systems are at risk for severe complications [4]. Toxoplasmosis is an illness that may be passed from mother to child during pregnancy, putting the unborn child at risk for significant health problems, including stillbirth, miscarriage, or congenital impairments [5]. Transmission to the unborn child is most likely to occur in the third trimester of pregnancy in the case of primary infection in the woman, according to the research of Pinto-Ferreira et al. [6]. There are more than 211 million births yearly [7]. Without proper monitoring and preventative measures, these pregnancies might end in the death of the mother or unborn child or neurological or cognitive impairments for the child [8,9]. The parasite *Toxoplasma gondii* has been associated with prenatal transmission of congenital toxoplasmosis [10,11]. The mother-to-child transmission happens when the woman is pregnant.

*Toxoplasma gondii* infection is managed by elimination after the first infection through cell-mediated immunity acquired by immunocompromised patients [12,13]. Bradyzoites of *Toxoplasma gondii* will become encysted in a host’s heart, liver, kidney, brain or skeletal muscle, and due to the high affinity of *Toxoplasma gondii* for nerve cells, cysts are also detected in the brain [10]. Pregnant women should avoid coming into contact with cat litter and dirt, which may contain oocysts of *Toxoplasma gondii*, in addition to significant risk from ingesting food contaminated with the parasite [14]. The significance of risk-control methods in using a “One-Health” approach to eradicating Toxoplasma infections cannot be emphasized. This is because swallowing tissue cysts has been less of a concern than the possibility of environmental transmission of oocysts [14,15]. Oocyst consumption, passed in infected cats’ feces, is the most common transmission route of Toxoplasmosis in the general population. Toxoplasmosis is an infection that may be passed from mother to child during pregnancy (vertical transmission), and transmission is most likely to occur in the third trimester of pregnancy [16]. Transplacental transmission in acute *Toxoplasma gondii* infection has been associated with many adverse outcomes, including stillbirths, congenital malformations, and deaths in newborns in immunocompromised mothers [11]. Local and systemic brain lesions, pneumonia, chorioretinitis, systemic infection, coma, and even death are all possible results [17]. Although a tight association between man and cats, the reservoir for the disease, has been widely recognized, the extent of the burden of *Toxoplasma gondii* in the WHO Eastern Mediterranean Region population is unknown [17]. This study aimed to perform a systematic literature review and meta-analysis to determine the pooled seroprevalence of *Toxoplasma gondii* among pregnant women in the WHO Eastern Mediterranean Region and determine maternal–fetal outcomes in pregnant women.

## 2. Materials and Methods

### 2.1. Search Strategies

The PRISMA (Preferred Reporting Items for Systematic Reviews and Meta-Analyses) criteria [18] were used in this study to ensure that no essential data were missed. Databases, including PubMed, SCOPUS, MEDLINE, and Index Medicus for the Eastern Mediterranean Region (IMEMR) were scoured for relevant articles. The search terms “*Toxoplasma gondii*” OR “*Toxoplasma* infection” AND Pregnancy OR “Pregnant women” ORpregnan* OR Antenatal or Prenatal OR Gravidity OR Parturition OR Maternal AND Afghanistan OR Bahrain OR Djibouti OR Egypt OR Iran OR Iraq OR Jordan OR Kuwait OR Lebanon OR Libya OR Morocco OR Pakistan OR Palestinian OR Qatar OR “Saudi Arabia” OR Somalia OR “South Sudan” OR Sudan OR Syria OR Tunisia OR “United Arab Emirates” OR Yemen were performed till 30 January 2023 with eligible papers published in the English language. Articles were included if they met the following criteria: if the study was conducted in one of the designated WHO Eastern Mediterranean Region countries, and if it was a cross-sectional, case-control, and cohort-based population study.

### 2.2. Eligibility Criteria

#### 2.2.1. Study Design

This study is a systematic review and meta-analysis, with a quantitative research design. The study used original articles with cross-sectional, cohort, and case-control designs that examined *Toxoplasma gondii* infection in pregnant women in the WHO Eastern Mediterranean Region. However, case reports and case series reports were not included in this study; any study with a sample size less than 10 was removed.

#### 2.2.2. Study Setting

Only studies on *Toxoplasma gondii* infection in pregnant women in Eastern Mediterranean countries were included. Only studies from Afghanistan, Bahrain, Djibouti, Egypt, Iran, Iraq, Jordan, Kuwait, Lebanon, Libya, Morocco, Pakistan, Palestine, Qatar, Saudi Arabia, Somalia, South Sudan, Sudan, Syria, Tunisia, United Arab Emirates and Yemen were included. Studies performed outside these countries were excluded.

### 2.3. Time Frame, Years Considered, and Language

There was no restriction on the time frame or years of publication of the articles to be included; all the articles published irrespective of the year of publication were included. However, the database search was carried out on 30 January 2023. Furthermore, a language restriction to the English language was applied in the search as there was no translation from French or other individual countries’ native languages where the primary studies are carried out. This was discussed under the limitations of the study.

### 2.4. Publication Type and Status of the Included Studies

**Figure 1:**
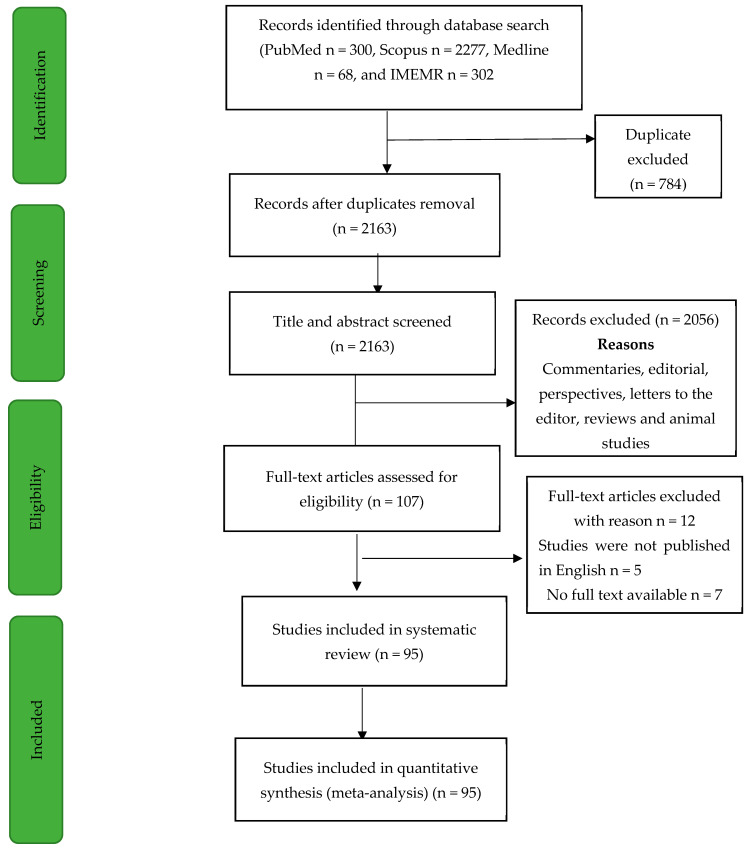
Flowchart of the screening process. Flowchart shows the screening process used, which follows PRISMA (Preferred Reporting Items for Systematic Reviews and Meta-Analyses) criteria.

The included articles were those that had undergone peer review and have been published, and the complete reports were available for reuse with acknowledgment of the authors by referencing. The designs of the studies that were included may have been either cohort, cross-sectional, or case-control. The study did not consider sources such as editorials, reviews, opinions, letters to the editor, and others, or studies that based their conclusions on secondary sources. *Toxoplasma gondii seropositivity* had to be detected using either ELISA or other means to detect immunoglobulins (Ig) in the serum samples. Studies were rejected if they did not involve pregnant women. The prevalence of *Toxoplasma gondii* infection in pregnant women was the primary outcome of concern; the secondary outcomes were maternal death, bleeding and miscarriage, while the fetal outcomes were low birth weight, preterm delivery and fetal mortality. Figure 1 depicts the selection procedure.

#### 2.4.1. Study Population

The population included in the study were pregnant women from the WHO Eastern Mediterranean Region that had been studied for *Toxoplasma gondii* infection. Studies that determined *Toxoplasma gondii* infection among pregnant women in the WHO Eastern Mediterranean Region that used cross-sectional, case-control, and cohort design that had more than ten samples were included as they reduced the risk of bias, as compared to studies that have less than ten samples or case reports and case series, which could introduce systematic bias. It has been stated that for a prevalence study, when determining pooled prevalence, care should be applied not to include studies that are subject to systematic bias; one of such biases is the inclusion of studies that have low sample sizes which could have a high positive outcome and thereby increase the heterogeneity in the study.

#### 2.4.2. Quality Assessment

The Joanna Briggs Institute (JBI) updated critical evaluation checklist was used to evaluate the study’s quality [19]. Disagreements about which articles should be included were settled by consensus per the JBI criteria. Research has shown that JBI is superior to other tools for determining prevalence in systematic reviews, and its results are straightforward to comprehend [20]. *Toxoplasma gondii* infection in pregnant women is a topic of this systematic review, and the criteria for using the JBI tools were satisfied. Each of the included articles was subjected to the eight-item checklist. For each “yes”, one point was added to the score. There is a range of zero to eight, from which the ultimate score for each study may be determined.

#### 2.4.3. Screening Processing

Web-based Endnote was used to screen the search articles; the duplicate article detection function in Web based Endnote was applied to the retrieved articles. After the titles and abstracts of all the remaining articles were reviewed, only the ones that made it through both were transferred to Excel. The data extraction phase was carried out.

#### 2.4.4. Data Extraction Process

Information such as the research’s initial author, year of publication, time of participant recruitment, country, year of study, study design, sample size, the sample tested for *Toxoplasma gondii* infection, and the number of individuals who tested positive for the infection were all extracted from the studies. The incidence of *Toxoplasma gondii* seropositive complications during pregnancy, such as diarrhea, jaundice and maternal mortality, was also measured as a secondary outcome. The incidence of adverse pregnancy outcomes such as preterm delivery, stillbirth, low birth weight and neonatal jaundice in mothers with *Toxoplasma gondii seropositive* was also documented.

#### 2.4.5. Data Analysis

OpenMeta Analyst was used for all statistical tests. The study’s pooled prevalence of *Toxoplasma gondii* infection was estimated using a random-effect model. For this reason, we used a random-effect model to examine highly heterogeneous data (I^2^ > 50%), the Cochran’s Q test was used in situations where the I^2^ < 40%. We also performed publication bias analyses to see our predictions’ robustness when only high-quality research was included. The test developed by Egger was used to examine publication bias, and the symmetry of counter-enhanced funnel plots was used. The likelihood of substantial publication bias was considered at a *p*-value of less than 0.10. A prevalence meta-regression study was also carried out to examine the prevalence concerning factors such as geographical location, presence or absence of symptoms, and other clinical features. Forest plots for all the important factors were made. The effect size, the rate of occurrence, and a confidence range of 95% were calculated for each study. OpenMeta Analyst returns numbers as decimal fractions; multiplication by 100 was carried out to obtain the percentage equivalent. As a consequence, the final results are expressed as a percentage.

## 3. Results

### 3.1. Description of the Studies

**Figure 2:**
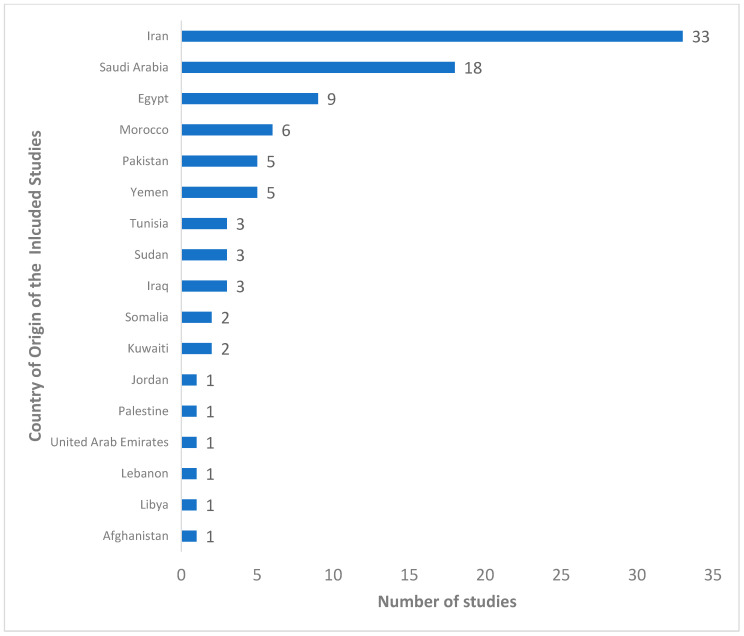
Distribution of the included studies by country of study origin. The figure above shows the number of studies found in each of the countries in the WHO Eastern Mediterranean region. The figure shows the number of studies from countries in descending order.

**Figure 3:**
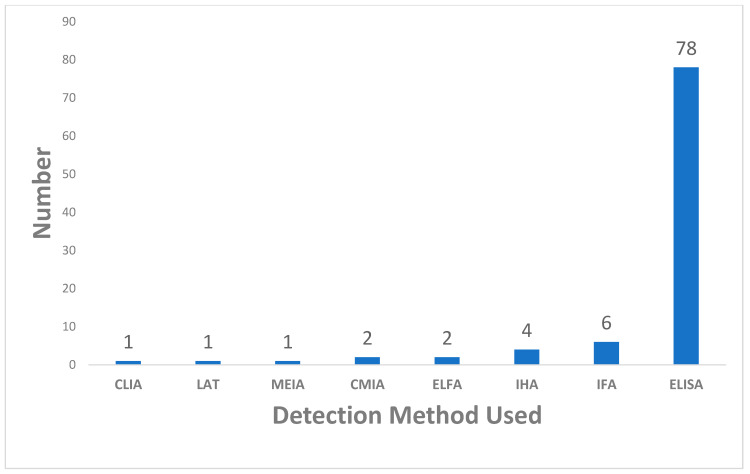
Distribution of the included studies by the method used. The figure shows the number of studies that used various methods to detect the antibodies of *Toxoplasma gondii.* The figure shows studies based on the method of detection of antibodies in ascending order.

Out of the 2947 articles retrieved from four databases (PubMed, Scopus, Medline, and IMEMR), 784 duplicated articles were removed, while 2163 articles were screened. At the abstract and title screening level, 2056 articles were removed. The remaining 107 were examined; 12 articles were further removed, 7 articles had no full text available, and 5 articles were removed as they were reported in a language other than English. The data from the 95 articles were extracted for further analysis and synthesis. The flow chart of the screening process is presented in Figure 1 above. The included studies’ characteristics are shown in Supplementary Table S1, while Figure 2 shows the distributions of the number of studies from the WHO Eastern Mediterranean Region. Of the 95 studies included, most (93) were cross-sectional studies, while 2 were case-control studies. Figure 2 shows the distribution of the included studies by country of origin. The 95 studies were carried out in eighteen countries; the highest number of studies, 33, was from Iran, followed by Saudi Arabia with 18 studies, 9 studies from Egypt, six studies from Morocco, 5 studies each from Pakistan and Yemen, 3 studies each from Tunisia, Sudan, and Iraq, 2 studies each from Somalia and Kuwaiti and 1 study each from other countries. Figure 3 shows the included studies based on the diagnostic method used. Of the 95 included studies, 78 studies used enzyme-linked immunosorbent assay (ELISA), six studies each used an indirect immunofluorescent test (IFAT) and indirect hemagglutination assay (IHA), 2 studies each used chemiluminescent microparticle (CMIA) and enzyme-linked fluorescent assay techniques (ELFA). In contrast, 1 study each used chemiluminescent immunoassay (CLIA), latex agglutination test (LAT), and microparticle enzyme immunoassay (MEIA) as shown in Figure 3. The quality assessment of the included studies is shown in supplementary data Table S1; they ranged from 6 to 8 on a scale of 8.

### 3.2. Prevalence of Toxoplasma gondii Infection in Pregnant Women in WHO Eastern Mediterranean Region

**Figure 4:**
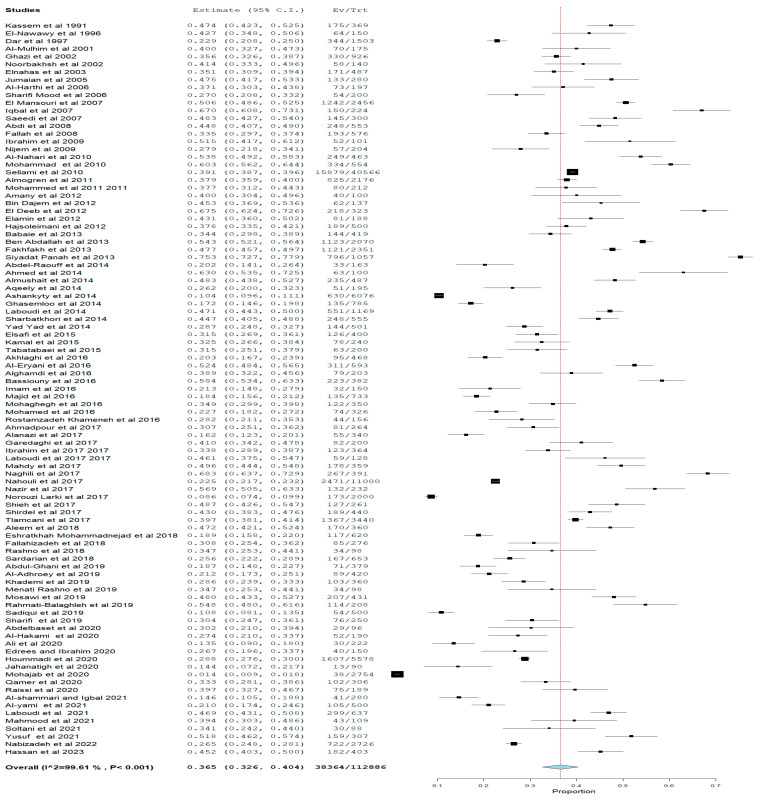
Overall Prevalence of *Toxoplasma gondii* in pregnant women in WHO Eastern Mediterranean Region. Figure shows the overall estimate of the *Toxoplasma gondii* seropositive (IgM or IgG positivity). The pooled seropositive prevalence was 36.5% with significant (*p* < 0.05) heterogeneity of 99.5%. The Ev in the plot means the number of events, while Tr means total numbers.

**Figure 5:**
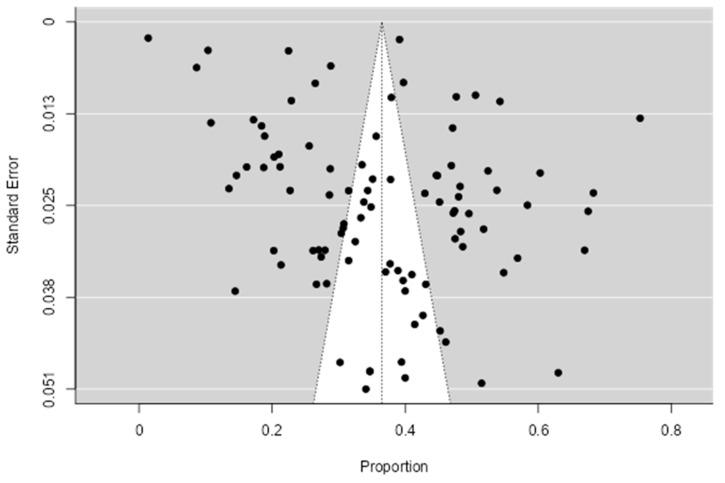
Funnel plot of the included articles. The figure presents the funnel plot for the included studies. The blank points show the distribution of the studies included and how they are spread from the center of the funnel. The cluster of studies at the top of the funnel shows they have smaller standard errors, while those at the bottom have large standard error. The funnel plot shows a mix of studies of small- and large-size samples.

To determine the pooled seroprevalence of *Toxoplasma gondii*, a total of 112,886 pregnant women from 95 studies from eighteen WHO Eastern Mediterranean Region countries were included in the quantitative analysis (meta-analysis). In total, 38,364 pregnant women were positive for anti-*Toxoplasma gondii* with a pooled seroprevalence of 36.5% (95%CI: 32.6–40.4) with a median value of 35.64%, the 25% and 75% interquartile ranges were 26.67% and 47.22%, with range values of 1.38–75.30% and 99.61% heterogeneity, indicating variability in the included studies as shown in Figure 4. The funnel plot shows a mix of studies of small- and large-size samples, as shown (Figure 5), and the Egger’s regression asymmetry test (*p* = 0.014) and Kendall’s Tau test (*p* = 0.524) shows publication bias.

**Figure 6:**
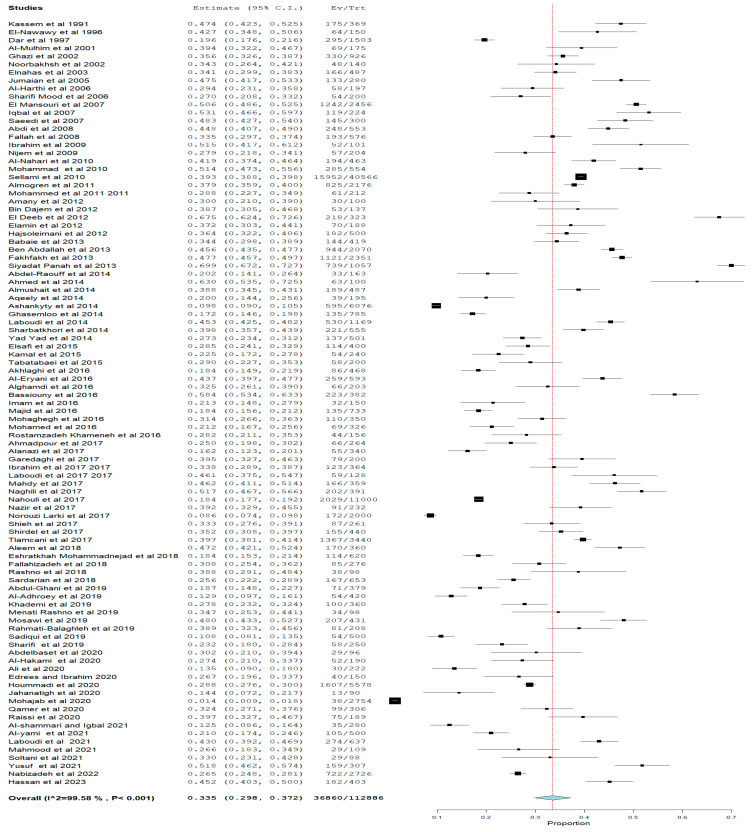
IgG Toxoplasma gondii Seropositive Prevalence in Pregnant Women in WHO Eastern Mediterranean Region. The figure shows the estimate of the Toxoplasma gondii IgG seropositive, the cumulative independent study IgG seroprevalences. The seropositive prevalence was 33.5% with significant (*p* < 0.05) heterogeneity of 99.6%. Ev means the number of events, while Tr means the total numbers.

**Figure 7:**
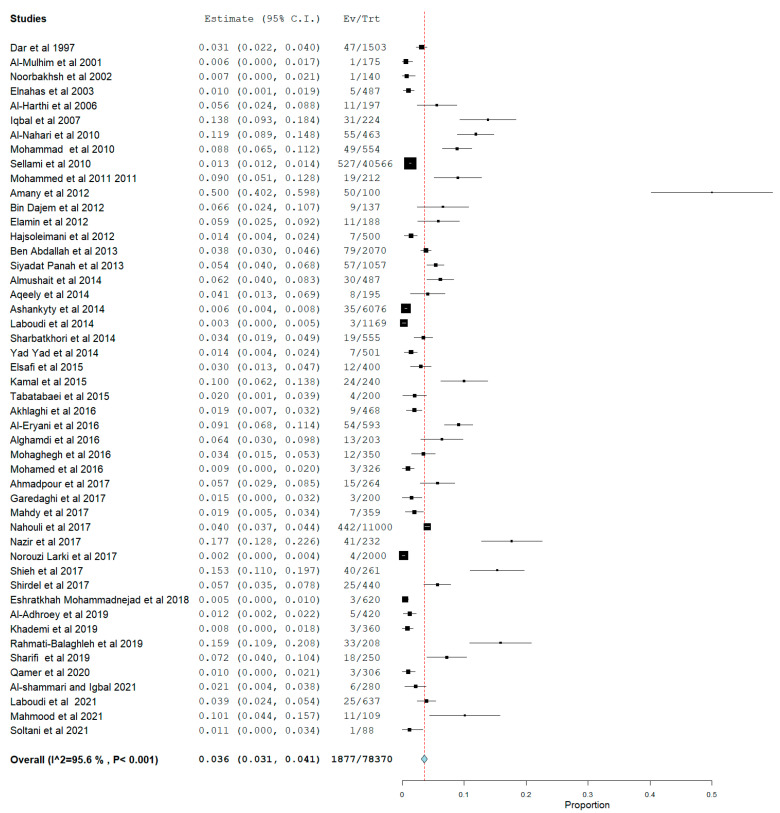
Prevalence of *Toxoplasma gondii* IgM Seropositive in Pregnant Women in WHO Eastern Mediterranean Region. Figure shows the *Toxoplasma gondii* IgM seropositivity estimate, which is the cumulative independent study IgM seroprevalence. The seropositive prevalence was 3.6% with significant (*p* < 0.05) heterogeneity of 95.6%. Ev means the number of events, while Tr means total numbers.

**Figure 8:**
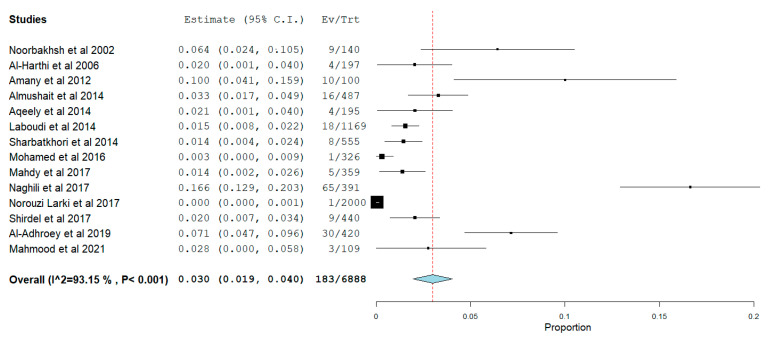
Prevalence of Both IgM and IgG Seropositivity of *Toxoplasma gondii* in Pregnant Women in WHO Eastern Mediterranean Region. The figure presents the estimate of the *Toxoplasma gondii* IgG and IgM seropositivity, the cumulative independent study seroprevalence. The seropositive prevalence was 3.0% with significant (*p* < 0.05) heterogeneity of 93.2%. Ev means number of events, while Tr means total numbers.

Figure 6, Figure 7 and Figure 8 show the estimated pooled seroprevalence in pregnant women based on antibody outcomes. All the 95 studies reported IgG *Toxoplasma gondii* infection with a rate of 33.5% (95%CI: 29.8–37.2) pooled seroprevalence and a median value of 33.51%; the 25% and 75% interquartile ranges were 25.00% and 43.01%, with range values of 1.38–69.92%, from 36,860 out of a population of 112,886 pregnant women. IgM pooled seroprevalence was 3.6% (95%CI: 3.1–4.1) with a median value of 3.62%; the 25% and 75% interquartile ranges were 0.0% and 3.81% with range values of 0.20–17.47% from 1877 out of 78,370 pregnant women in 48 studies. Both IgM and IgG pooled seroprevalence was 3.0% (95%CI: 1.9–4.4) with a median value of 2.05%; the 25% and 75% interquartile ranges were 0.01% and 6.13% with range values of 0.05–16.62% from 183 out of a population of 6888 pregnant women in 14 studies.

Table 1 reports subgroup analysis of the *Toxoplasma gondii* pooled seroprevalence by year of the study, countries, method, and recruitment settings.

The pooled seroprevalence of *Toxoplasma gondii* seropositivity in the study showed that 41.1%, 41.3%, 32.2% and 34.8% were recorded in studies carried out in <2010, 2010–2015, 2016–2020, and >2020, respectively.

Based on the country of origin of the study, Somalia with *Toxoplasma gondii* pooled seroprevalence of 48.3% (95%CI: 41.8–54.8) from 341 out of 710 pregnant women population in two studies was the highest, followed by Afghanistan (48.0% (95%CI: 43.3–52.7). The lowest seroprevalence was recorded in Lebanon (22.5%) and United Arab Emirates (22.9%). The pooled seroprevalence of *Toxoplasma gondii* found in pregnant women was 46.6% in Egypt, 35.2% in Iran, 29.2% in Pakistan, 30.7% in Saudi Arabia, and 43.1% in Morocco. The pooled seroprevalence found in other countries is presented in Table 1.

The pooled seroprevalence of *Toxoplasma gondii* based on the method used in pregnant women is as follows. The pooled seroprevalence based on the diagnostic method used, ELFA was 67.3%, which showed the highest occurrence; ELISA showed 35.9% pooled seroprevalence, CLIA, CMLA, IFAT, IHA, LAT, and MEIA methods reported 26.5%, 35.9%, 30.9%, 47.5%, 10.8%, 41.9%, 47.2% and 40.0%, respectively, as shown in Table 1.

There was no significant pooled seroprevalence of *Toxoplasma gondii* infection of pregnant women based on their recruitment setting. The pregnant women recruited in the Antenatal clinic (ANC) showed 34.1%, while those from Non-Antenatal clinic (Non-ANC) showed 38.1% pooled seroprevalence, as presented in Table 1.

### 3.3. Determinant Factors Associated with Toxoplasma gondii Seropositivity in Pregnant Women from WHO Eastern Mediterranean Region

The five determinant factors mostly reported in our study were contact with a cat, unprocessed milk, eating raw or undercooked meat, and eating unwashed raw vegetables. Of the 95 studies examined, 28 of them explored contact with cats as a risk factor, 34.8% (1281 of 3389) of seropositive *Toxoplasma gondii* pregnant women reported contact with a cat, 32.9% (174 of 1765) drank unprocessed milk, 43.7% (1311 of 3101) ate raw or undercooked meat, and 40.8% (715 of 1683) ate unwashed raw vegetables, as shown in Table 2.

### 3.4. Maternal and Fetal Outcomes Associated with Toxoplasma gondii Seropositive in Pregnant Women from WHO Eastern Mediterranean Region

*Toxoplasma gondii* seropositive women was associated with a history of miscarriage (36.1%% (112 of 300)), stillbirth (25.7% (111 of 375)), intrauterine fetal death (1.9% (3 of 157)) and premature delivery (20.1% (96 of 362)) as shown in Table 2.

The study found *Toxoplasma gondii* antibody positivity in pregnant women to be associated with fetal-associated outcomes such as low birth weight (29.3% (77 of 350)) and and neonatal death (3.2% (6 of 183), as shown in Table 3.

## 4. Discussion

*Toxoplasma gondii* infection is implicated in pregnancy for women who may be immunocompromised globally [21]. The overall pooled prevalence of *Toxoplasma gondii* seropositivity in pregnant women in the WHO Eastern Mediterranean Region was 36.5% (95%CI: 32.6–40.4) with 99.61% heterogeneity, the *Toxoplasma gondii* IgM positivity was 3.6%’ (95%CI: 3.1–4.1), which may be indicative of acute *Toxoplasma gondii* infection, while the pooled IgG positivity was 33.5%; (95%CI: 29.8–37.2), which may be indicative of chronic *Toxoplasma gondii* infection. The publication bias was significant based on Egger’s asymmetry test (*p* = 0.014) and Kendall’s Tau test (*p* = 0.524). The identified risk factors in the study were contact with cats, eating raw unwashed vegetables, and uncooked meat. The maternal–fetal outcomes associated with *Toxoplasma gondii* positivity found in the study were stillbirth, abortion or miscarriage, premature delivery and low birth weight.

Due to the high heterogeneity of the results, generalizing the result to the whole population needs to be done with care. Furthermore, as found in this study, most included studies were cross-sectional studies, which cannot study risk factors and the contributions of *Toxoplasma gondii* positivity to the identified maternal–fetal outcomes. As is well known, prospective, cohort and case-control studies are more appropriate for studying associated risk factors and the results of a particular infection or condition [22,23].

The overall pooled prevalence of *Toxoplasma gondii* seropositivity in pregnant women of the WHO Eastern Mediterranean Region was 36.5%. This found pooled prevalence of *Toxoplasma gondii* seropositivity in pregnant women of WHO Eastern Mediterranean Region countries was higher than the 32.9% pooled prevalence of *Toxoplasma gondii* IgG infection reported in a global systematic review and meta-analysis in pregnant women that was reported by Bigna et al. [24]. Similarly, in the general population, a systematic review and meta-analysis carried out by Molan et al. [25] reported 25.7% pooled prevalence, which was also lower than what is reported in this study. However, the study by Molan et al. [25] was carried out in all populations. Comparing this study to a similar study by Rostami et al. [13] showed a seroprevalence of 33.8%, which was slightly lower than what is reported in this study.

The pooled prevalence of 36.5% reported in this study was lower than the 51.0% pooled prevalence of seropositive of *Toxoplasma gondii* reported in Africa by Dasa et al. [11] in pregnant women. Odeniran et al. [21] also reported a *Toxoplasma gondii* pooled prevalence of 45.4% in pregnant women in a study carried out in Nigeria. However, it was observed that several eligible articles were excluded.

The reported prevalence of IgM *Toxoplasma gondii* positivity of 3.6% was higher than the global pooled prevalence of 1.9%, as Bigna et al. [24] reported. Similarly, the pooled prevalence of IgG *Toxoplasma gondii* positivity in pregnant women in the WHO Eastern Mediterranean Region at 33.5% was slightly higher than the global pooled prevalence of 32.9% reported by Bigna et al. [24]. The findings in this study were higher than the IgM *Toxoplasma gondii* positivity reported by Molan et al. [25], who reported a global snapshot of *Toxoplasma gondii* seropositivity.

The pooled prevalence reported in our study was a bit higher than that reported in the Karshima and Karshima [26] study, which documented a pooled prevalence of 32.9% in the general population. However, the pregnant women subgroup analysis of *Toxoplasma gondii* infection by the study by Karshima and Karshima [26] showed a pooled prevalence of 40.25%.

In terms of the yearly *Toxoplasma gondii* seropositivity, the highest prevalence was reported in studies carried out prior to 2010, which reported 41.1% pooled prevalence. D’Mello-Guyett et al. [27] established a link between water, sanitation and hygiene (WASH) and water borne infection, which had been established earlier. Duedu et al. [28] and Parvin et al. [29] assert that the implementation of WASH programs was in the elementary phase before 2010, which made infections associated with WASH to be common; however, as the implementation progressed, a decline in WASH-related infections was reported, which could also be the case in this finding of a higher prevalence at that period.

The report of this study shows that contact with cats is a major risk factor that could be the source of oocyst contamination in the environment and hence play an important role in *Toxoplasma gondii* epidemiology. Anti-*Toxoplasma gondii* antibodies are substantially more prevalent in pregnant women exposed to cats than those who are not. As a result, some of the cats were probably already shedding the oocyst when they came into contact [30]. Furthermore, eating raw vegetables and uncooked meat was also found to be a main factor that was also independently reported by some studies within and outside Africa [31]. In a study carried out by Retmanasari et al. [32], a link was established between cat population density, contact with raw meat, and unclean water; this showed the importance of water, sanitation and hygiene (WASH) in the prevention of infections that are associated with access to potable water, a clean environment, and excellent hygiene practices.

The maternal–fetal outcomes found to have been associated with *T. gondii* seropositivity in our study were stillbirth, abortion, miscarriages, premature delivery and low birth weight, which has been documented in the literature [29,33,34,35,36]. Furthermore, a recent study by Parvin et al. [29] reported that abortion was a possible maternal clinical outcome in *Toxoplasma gondii* infection in pregnancy. Fochi et al. [33] established a correlation between prematurity, low birth weight and maternal toxoplasmosis in pregnancy. Parvin et al. [29] also established the same in their recent study. Congenital toxoplasmosis is also established in babies born to pregnant women with *Toxoplasma gondii* infection with HIV and other immune-compromising diseases [37].

## 5. Limitations

Most of the included studies did not report the IgG avidity, which would have enabled us to report the actual infection status in the study. Still, most reported the IgM or IgG state without reference to the IgG avidity, which enables Toxoplasma gondii positivity to develop into an acute or chronic infection. Due to high heterogeneity, the interpretation and application of the findings to the general population should be done cautiously. Furthermore, there were no appropriate data from the included studies to perform risk factor analysis; most of the studies only reported proportional data for the positive as most of them were designed as cross-sectional studies, which are not appropriate for risk factor analysis.

## 6. Conclusions

*Toxoplasma gondii* seroprevalence was high among pregnant women in the WHO Eastern Mediterranean Region and was shown to be associated with risks: contact with cats, eating raw vegetables, and raw or uncooked meat. As such, hygienic practices should be improved to reduce the transmission from those sources. The maternal–fetal outcomes found in this study were stillbirth, abortion, miscarriages, premature delivery and low birth weight. Because of the poor sociopolitical and living situations in the WHO Eastern Mediterranean Region and the lack of knowledge, thousands of immune-compromised persons and pregnant women are at risk of contracting toxoplasmosis. There is a potential for more severe implications for public health and economic expenses in the WHO Eastern Mediterranean Region. It is important as Toxoplasma gondii is found in humans, the food chain and the environment. One-Health preventative measures should be implemented. These responsibilities must be assigned to the Ministry of Health and non-governmental groups so that the burden on society and the economy may be alleviated and congenital transmission can be stopped. The study also found that the pooled prevalence between 2020 and 2022 demonstrates that the infection rate is still high. From the findings of this study, the following is therefore recommended. The deficiency found in most of the included studies was in the study design. Most studies used a cross-sectional design that is ineffective in assessing risk factors and clinical outcomes. Therefore, an appropriate study design that can determine risk factors and clinical outcomes is needed in Eastern Mediterranean countries to ascertain the real risk factors and clinical outcomes associated with *Toxoplasma gondii* seropositivity in pregnant women in the WHO Eastern Mediterranean Region. Therefore, *Toxoplasma gondii* should be included in pre-pregnancy screening, or screening very early in pregnancy to reduce negative outcomes from its vertical transmission. The One-Health approach should be applied to control Eastern Mediterranean countries since a known animal reservoir is implicated in transmitting *Toxoplasma gondii*. WASH must be applied to reduce transmission.

## Figures and Tables

**Table 1:** Subgroup analysis of Prevalence of *Toxoplasma gondii* Seropositivity in Pregnant Women in WHO Eastern Mediterranean Region.

Subgroup	Number of Studies	n/N	Seroprevalence (%)	95% CI	
				Lower Limit	Upper Limit
Year Range					
<2010	17	3559/8841	41.1	35.1	47.0
2010–2015	26	23,498/61,987	41.3	34.3	48.4
2016–2020	44	9726/37,008	32.2	27.0	37.4
>2020	8	1581/5050	34.8	25.9	43.7
Country					
Afghanistan	1	207/431	48.0	43.3	52.7
Egypt	9	890/1856	46.6	36.6	56.7
Iran	33	5167/15,972	35.2	28.7	41.7
Iraq	3	163/471	34.4	26.4	42.4
Jordan	1	133/280	47.5	41.7	53.3
Kuwait	2	191/504	40.8	10.5	92.0
Lebanon	1	2471/11,000	22.5	21.7	23.2
Libya	1	175/369	47.4	42.3	52.5
Morocco	6	5125/13,408	43.1	34.6	51.6
Pakistan	5	521/2047	29.2	14.5	43.8
Palestine	1	57/204	27.9	21.8	34.1
Saudi Arabia	18	3273/16,092	30.7	23.4	38.0
Somalia	2	341/710	48.3	41.8	54.8
Sudan	3	285/838	32.8	21.0	44.5
Tunisia	3	18,123/44,987	47.0	37.4	56.6
United Arab Emirates	1	344/1503	22.9	20.8	25.0
Yemen	5	898/2214	39.1	23.2	55.0
Diagnostic used					
CLIA	1	722/2726	26.5	24.8	28.1
CMIA	2	1493/3840	35.9	27.9	44.0
ELFA	2	368/547	67.3	63.3	71.2
ELISA	78	33,620/99,601	35.9	31.5	40.3
IFA	6	817/2842	28.7	17.8	39.6
IHA	4	1104/2795	41.9	36.4	47.4
LAT	1	170/360	47.2	42.1	52.4
MEIA	1	70/175	40.0	32.7	47.3
Recruitment Settings					
ANC	37	25,771/74,425	34.1	27.4	40.8
NON-ANC	38	12,593/38,461	38.1	33.3	42.9

Key: % = percentage, CI = confidence interval, ANC = Anti-natal clinic, n = number positive for the event, N = total number examined, Enzyme-Linked Immunosorbent Assay (ELISA), Indirect Immunofluorescent (IFA), Indirect Hemagglutination Assay (IHA), Chemiluminescent Microparticle (CMIA), Enzyme-Linked Fluorescent Assay (ELFA), Chemiluminescent Immunoassay (CLIA), Latex Agglutination Test (LAT) and Microparticle Enzyme Immunoassay (MEIA).

**Table 2:** Determinant Factors Associated with *Toxoplasma gondii* Seropositivity in Pregnant Women in WHO Eastern Mediterranean Region.

Risk Factors	Number of Studies	Sample Size	Number Positive	Pooled Prevalence (%)	95%CI	Chi-Square	*p*-Value
Contact with cat	28	3389	1281	34.8	23.7–46.0	14.5	0.004
Dinking of unprocessed milk	11	1765	174	32.9	17.4–48.4	11.3	0.001
Eating raw or uncooked meat	24	3101	1311	43.7	27.6–59.7	9.6	0.02
Eating unwashed raw vegetable	13	1683	715	40.8	16.7–65.0	15.8	0.001

CI = confidence interval. The table presents factors associated with *Toxoplasma gondii* seropositive. The associated factors identified were contact with cats, drinking unprocessed milk, eating raw or undercooked meat, and unwashed raw vegetables.

**Table 3:** Fetal and Neonatal Outcomes Associated with *Toxoplasma gondii* positivity in Pregnant Women in WHO Eastern Mediterranean Region.

Maternal–Fetal Outcomes	Number of Studies	Sample Size	Number Positive	Pooled Prevalence (%)	95%CI	Chi-Square	*p*-Value
**Fetal Outcome**							
History of abortion	22	2990	868	32.5	22.7–42.3	18.8	0.0001
History of miscarriage	2	300	112	36.1	21.8–50.4	3.6	0.09
History of stillbirth	4	375	111	25.7	3.5–47.8	6.8	0.002
Intrauterine fetal death	2	157	3	1.9	0.02–4.5	0.9	0.87
Premature delivered	4	362	96	20.1	1.7–41.7	9.74	0.003
**Neonatal Associated** **Outcome**							
Low birth weight	2	350	77	29.3	24.1–82.7	10.42	0.001
Neonatal death	2	183	6	3.2	0.7–5.8	0.61	0.76
Congenital abnormalities	6	725	17	1.7	0.2–3.2	1.65	0.34

CI = confidence interval. The table presents maternal and fetal outcomes of *Toxoplasma gondii* seropositivity which were history of abortion, history of miscarriage, history of stillbirth, premature delivery and low birth weight.

## Data Availability

The data used for this study is attached as a supplementary Excel file.

## Supplementary Materials

The following supporting information can be downloaded at: https://www.mdpi.com/article/10.3390/pathogens12091157/s1. Table S1: Study Characteristics and JBI Quality Assessment Score. File S1: Raw Data.

## References

[1] AAli S., Amjad Z., Khan T.M., Maalik A., Iftikhar A., Khan I., Ahmed H. (2020). Occurrence of *Toxoplasma gondii* antibodies and associated risk factors in women in selected districts of Punjab province, Pakistan. Parasitology.

[2] Rashno M.M., Fallahi S., Arab-Mazar Z., Dana H. (2019). Seromolecular assess of *Toxoplasma gondii* infection in pregnant women and neonatal umbilical cord blood. EXCLI J..

[3] Bigna J.J., Tochie J.N., Tounouga D.N., Bekolo A.O., Ymele N.S., Youda E.L., Sime P.S., Nansseu J.R. (2020). Global, regional, and country seroprevalence of *Toxoplasma gondii* in pregnant women: A systematic review, modelling and meta-analysis. Sci. Rep..

[4] Mahmood M.T., Kahya H.F.H. (2021). Serological study of torch complex in pregnant women with an obstetric history in mosul city, iraq. Curr. Trends. Immunol..

[5] Elamin M.H., Al-Olayan E.M., Omer S.A., Alagaili A.N., Mohammed O.B. (2012). Molecular detection and prevalence of *Toxoplasma gondii* in pregnant women in Sudan. African J. Microbiol. Res..

[6] Pinto-Ferreira F., Caldart E.T., Pasquali A.K.S., Mitsuka-Breganó R., Freire R.L., Navarro I.T. (2019). Patterns of Transmission and Sources of Infection in Outbreaks of Human Toxoplasmosis. Emerg. Infect. Dis..

[7] Plan International (2022). 10 Things You Should Know for World Contraception Day|Plan International UK. https://plan-uk.org/blogs/10-things-you-should-know-for-world-contraception-day.

[8] Mboera L.E.G., Kishamawe C., Kimario E., Rumisha S.F. (2019). Mortality Patterns of Toxoplasmosis and Its Comorbidities in Tanzania: A 10-Year Retrospective Hospital-Based Survey. Front. Public Health.

[9] Mendez O.A., Koshy A.A. (2017). Toxoplasma gondii: Entry, association, and physiological influence on the central nervous system. PLoS Pathog..

[10] Cerutti A., Blanchard N., Besteiro S. (2020). The Bradyzoite: A Key Developmental Stage for the Persistence and Pathogenesis of Toxoplasmosis. Pathogens.

[11] Dasa T.T., Geta T.G., Yalew A.Z., Abebe R.M., Kele H.U. (2021). Toxoplasmosis infection among pregnant women in Africa: A systematic review and meta-analysis. PLoS ONE.

[12] Raissi V., Taghipour A., Navi Z., Etemadi S., Sohrabi Z., Sohrabi N., Getso M., Shamsaei S., Karami M.F., Raiesi O. (2020). Seroprevalence of *Toxoplasma gondii* and *Toxocara* spp. infections among pregnant women with and without previous abortions in the west of Iran. J. Obstet. Gynaecol. Res..

[13] Rostami A., Riahi S.M., Gamble H.R., Fakhri Y., Shiadeh M.N., Danesh M., Behniafar H., Paktinat S., Foroutan M., Mokdad A.H. (2020). Global prevalence of latent toxoplasmosis in pregnant women: A systematic review and meta-analysis. Clin. Microbiol. Infect..

[14] Tenter A.M. (2009). *Toxoplasma gondii* in animals used for human consumption. Mem. Inst. Oswaldo Cruz..

[15] Sinha S., Sehgal A., Kaur U., Sehgal R., Parija S.C., Chaudhury A. (2022). Toxoplasmosis. Textbook of Parasitic Zoonoses.

[16] Oyeyemi O.T., Oyeyemi I.T., Adesina I.A., Tiamiyu A.M., Oluwafemi Y.D., Nwuba R.I., Grenfell R.F.Q. (2020). Toxoplasmosis in pregnancy: A neglected bane but a serious threat in Nigeria. Parasitology.

[17] Alsammani M.A. (2016). Sero-epidemiology and risk factors for *Toxoplasma gondii* among pregnant women in Arab and African countries. J. Parasit. Dis. Off. Organ. Indian Soc. Parasitol..

[18] Moher D., Liberati A., Tetzlaff J., Altman D.G. (2010). Preferred reporting items for systematic reviews and meta-analyses: The PRISMA statement. Int. J. Surg..

[19] Joanna Briggs Institute (2022). Critical-Appraisal-Tools—Critical Appraisal Tools|JBI. https://jbi.global/critical-appraisal-tools.

[20] Munn Z., Moola S., Riitano D., Lisy K. (2014). The development of a critical appraisal tool for use in systematic reviews addressing questions of prevalence. Int. J. Health Policy Manag..

[21] Odeniran P.O., Omolabi K.F., Ademola I.O. (2020). Risk factors associated with seropositivity for *Toxoplasma gondii* in population-based studies among immunocompromised patients (pregnant women, HIV patients and children) in West African countries, Cameroon and Gabon: A meta-analysis. Acta Trop..

[22] Apuke O. (2017). Quantitative Research Methods: A Synopsis Approach. Arab. J. Bus. Manag. Rev. (Kuwait Chapter).

[23] Kothari J. (2004). Research Methodology (Methods and Techniques).

[24] Bigna J.J., Modiyinji A.F., Nansseu J.R., Amougou M.A., Nola M., Kenmoe S., Temfack E., Njouom R. (2020). Burden of hepatitis e virus infection in pregnancy and maternofoetal outcomes: A systematic review and meta-analysis. BMC Pregnancy Childbirth.

[25] Molan A., Nosaka K., Wang W., Hunter M. (2019). Global status of *Toxoplasma gondii* infection: Systematic review and prevalence snapshots. Trop Biomed..

[26] Karshima S.N., Karshima M.N. (2020). Human *Toxoplasma gondii* infection in Nigeria: A systematic review and meta-analysis of data published between 1960 and 2019. BMC Public Health.

[27] D’mello-Guyett L., Gallandat K., Bergh R.V.D., Taylor D., Bulit G., Legros D., Maes P., Checchi F., Cumming O. (2020). Prevention and control of cholera with household and community water, sanitation and hygiene (WASH) interventions: A scoping review of current international guidelines. PLoS ONE.

[28] Duedu K.O., Yarnie E.A., Tetteh-Quarcoo P.B., Attah S.K., Donkor E.S., Ayeh-Kumi P.F. (2014). A comparative survey of the prevalence of human parasites found in fresh vegetables sold in supermarkets and open-aired markets in Accra, Ghana. BMC Res. Notes.

[29] Parvin I., Das S.K., Ahmed S., Rahman A., Bin Shahid A.S.M.S., Shahrin L., Afroze F., Ackhter M.M., Alam T., Jahan Y. (2022). *Toxoplasma gondii* Infection Is Associated with Low Birth Weight: Findings from an Observational Study among Rural Bangladeshi Women. Pathogens.

[30] Marín-García P.-J., Planas N., Llobat L. (2022). *Toxoplasma gondii* in Foods: Prevalence, Control, and Safety. Foods.

[31] Hussain M.A., Stitt V., Szabo E.A., Nelan B. (2017). *Toxoplasma gondii* in the Food Supply. Pathogens.

[32] Retmanasari A., Widartono B.S., Wijayanti M.A., Artama W.T. (2017). Prevalence and Risk Factors for Toxoplasmosis in Middle Java, Indonesia. EcoHealth.

[33] Fochi M.M.L., Baring S., Spegiorin L.C.J.F., Vaz-Oliani D.C.M., Galão E.A., Oliani A.H., de Mattos L.C., de Mattos C.C.B. (2015). Prematurity and Low Birth Weight did not Correlate with Anti-*Toxoplasma gondii* Maternal Serum Profiles—A Brazilian Report. PLoS ONE.

[34] Hurt K., Kodym P., Stejskal D., Zikan M., Mojhova M., Rakovic J. (2022). Toxoplasmosis impact on prematurity and low birth weight. PLoS ONE.

[35] Mocanu A.G., Stoian D.L., Craciunescu E.L., Ciohat I.M., Motofelea A.C., Navolan D.B., Vilibic-Cavlek T., Stevanovic V., Nemescu D., Forga M. (2022). The Impact of Latent *Toxoplasma gondii* Infection on Spontaneous Abortion History and Pregnancy Outcomes: A Large-Scale Study. Microorganisms.

[36] Nayeri T., Sarvi S., Moosazadeh M., Amouei A., Hosseininejad Z., Daryani A. (2020). The global seroprevalence of anti-*Toxoplasma gondii* antibodies in women who had spontaneous abortion: A systematic review and meta-analysis. PLoS Neglected Trop. Dis..

[37] Megli C.J., Coyne C.B. (2022). Infections at the maternal–fetal interface: An overview of pathogenesis and defence. Nat. Rev. Microbiol..

