# Supplementary material for: Seroprevalence, Risk Factors and Maternal–Fetal Outcomes of Toxoplasma gondii in Pregnant Women from WHO Eastern Mediterranean Region: Systematic Review and Meta-Analysis
Source: Pathogens. 2023 Sep 12;12(9):1157. doi: 10.3390/pathogens12091157 (PMC10537388; doi:10.3390/pathogens12091157)
Supplement: Supplementary file 1 [file pathogens-12-01157-s001.zip › Supplementary Table S1_new.pdf]

Table S1: Study Characteristics and JBI Quality Assessment Score

| Author | Year | Country | Study Design | Diagnosis method | Total | Number of Case (IgM or IgG positive) | JBI Score Result |
|---|---|---|---|---|---|---|---|
| Kassem & Morsy [1] | 1991 | Libya | Cross-sectional | IHA | 369 | 175 | 8 |
| El-nawawy [2] | 1996 | Egypt | Cross-sectional | IHA | 150 | 64 | 7 |
| Dar et al [3] | 1997 | United Arab Emirates | Cross-sectional | ELISA | 1503 | 344 | 8 |
| Al-mulhim & Al-qurashi [4] | 2001 | Saudi Arabia | Cross-sectional | MEIA | 175 | 70 | 6 |
| Ghazi et al [5] | 2002 | Saudi Arabia | Cross-sectional | ELISA | 926 | 330 | 8 |
| Noorbakhsh et al. [6] | 2002 | Iran | Cross-sectional | ELISA | 140 | 58 | 8 |
| Elnahas et al [7] | 2003 | Sudan | Cross-sectional | ELISA | 487 | 171 | 8 |
| Jumaian [8] | 2005 | Jordan | Cross-sectional | IFAT | 280 | 133 | 6 |
| Al-harthi et al [9] | 2006 | Saudi Arabia | Cross-sectional | ELISA | 197 | 73 | 8 |
| Sharifi-mood et al. [10] | 2006 | Iran | Cross-sectional | IFA | 200 | 54 | 6 |
| Mansouri et al.[11] | 2007 | Morocco | Cross-sectional | ELISA | 2456 | 1242 | 7 |
| Iqbal & Khalid [12] | 2007 | Kuwaiti | Cross-sectional | ELFA | 224 | 150 | 7 |
| Saeedi & Marjani [13] | 2007 | Iran | Cross-sectional | ELISA | 300 | 145 | 8 |
| Abdi et al. [14] | 2008 | Iran | Cross-sectional | IFA | 553 | 248 | 8 |
| Fallah et al. [15] | 2008 | Iran | Cross-sectional | IFA | 576 | 193 | 8 |
| Ibrahim et al. [16] | 2009 | Egypt | Cross-sectional | ELISA | 101 | 52 | 8 |
| Nijem & Al-Amleh [17] | 2009 | Palestine | Cross-sectional | ELISA | 204 | 57 | 7 |
| Al-Nahari et al. [18] | 2010 | Yemen | Cross-sectional | ELISA | 463 | 249 | 7 |
| (Mohammad et al. [19] | 2010 | Saudi Arabia | Cross-sectional | ELISA | 554 | 334 | 8 |
| Sellami et al. [20] | 2010 | Tunisia | Cross-sectional | ELISA | 40566 | 15879 | 8 |

| | | | | | | | |
|---|---|---|---|---|---|---|---|
| Almogren [21] | 2011 | Saudi Arabia | Cross-sectional | IHA | 2176 | 825 | 8 |
| Mohammed [22] | 2011 | Iraq | Cross-sectional | ELISA | 212 | 80 | 8 |
| Amany & and Merwad [23] | 2012 | Egypt | Cross-sectional | IHA | 100 | 40 | 8 |
| Saad M. Bin Dajem & Almushait [24] | 2012 | Saudi Arabia | Cross-sectional | ELISA | 137 | 62 | 7 |
| El Deeb et al. [25] | 2012 | Egypt | Cross-sectional | ELFA | 323 | 218 | 8 |
| Maha H. Elamin [26] | 2012 | Sudan | Cross-sectional | ELISA | 188 | 81 | 6 |
| Hajsoleimani et al. [27] | 2012 | Iran | Cross-sectional | ELISA | 500 | 189 | 8 |
| Babaie et al. [28] | 2013 | Iran | Cross-sectional | ELISA | 419 | 144 | 8 |
| (Ben Abdallah et al. [29] | 2013 | Tunisia | Cross-sectional | ELISA | 2070 | 1123 | 7 |
| Fakhfakh et al. [30] | 2013 | Tunisia | Cross-sectional | ELISA | 2351 | 1121 | 8 |
| Panah Abolghasem Siyadat et al. [31] | 2013 | Iran | Cross-sectional | ELISA | 1057 | 796 | 8 |
| Abdel-Raouff & Elbasheir [32] | 2014 | Sudan | Cross-sectional | ELISA | 163 | 33 | 7 |
| Ahmed et al. [33] | 2014 | Egypt | Cross-sectional | ELISA | 100 | 63 | 7 |
| Almushait et al. [34] | 2014 | Saudi Arabia | Cross-sectional | ELISA | 487 | 235 | 8 |
| Aqeely et al. [35] | 2014 | Saudi Arabia | Cross-sectional | ELISA | 195 | 51 | 8 |
| Ashankyty [36] | 2014 | Saudi Arabia | Cross-sectional | ELISA | 6076 | 630 | 7 |
| Hadi et al. [37] | 2014 | Iran | Cross-sectional | ELISA | 785 | 135 | 8 |
| M Laboudi et al [38] | 2014 | Morocco | Cross-sectional | ELISA | 1169 | 551 | 8 |
| (Sharbatkhori et al. [39] | 2014 | Iran | Cross-sectional | ELISA | 555 | 248 | 7 |
| Yad Yad et al [40] | 2014 | Iran | Cross-sectional | ELISA | 501 | 144 | 8 |
| Elsafi et al. [41] | 2015 | Saudi Arabia | Cross-sectional | CMIA | 400 | 126 | 7 |

| | | | | | | | |
|---|---|---|---|---|---|---|---|
| Kamal et al. [42] | 2015 | Egypt | Cross-sectional | ELISA | 240 | 78 | 7 |
| Ahmadpour et al.[43] | 2015 | Iran | Cross-sectional | ELISA | 200 | 63 | 8 |
| Akhlaghi et al. [44] | 2016 | Iran | Cross-sectional | ELISA | 468 | 95 | 7 |
| Al-Eryani et al. [45] | 2016 | Yemen | Cross-sectional | ELISA | 593 | 311 | 8 |
| Alghamdi et al. [46] | 2016 | Saudi Arabia | Cross-sectional | ELISA | 203 | 79 | 7 |
| Bassiony et al. [47] | 2016 | Egypt | Cross-sectional | ELISA | 382 | 223 | 8 |
| Imam et al. [48] | 2016 | Saudi Arabia | Cross-sectional | ELISA | 150 | 32 | 7 |
| Majid et al. [49] | 2016 | Pakistan | Cross-sectional | IFA | 733 | 135 | 8 |
| (Mohaghegh et al. [50] | 2016 | Iran | Cross-sectional | ELISA | 350 | 122 | 7 |
| Mohamed et al. [51] | 2016 | Saudi Arabia | Cross-sectional | ELISA | 326 | 74 | 8 |
| Rostamzadeh Khameneh et al. [52] | 2016 | Iran | Cross-sectional | ELISA | 156 | 44 | 7 |
| (Ahmadpour et al. [53] | 2017 | Iran | Cross-sectional | ELISA | 264 | 81 | 8 |
| Alanazi et al. [54] | 2017 | Saudi Arabia | Cross-sectional | ELISA | 340 | 55 | 7 |
| Garedaghi & Firozivand [55] | 2017 | Iran | Cross-sectional | ELISA | 200 | 82 | 8 |
| Ibrahim et al. [56] | 2017 | Egypt | Cross-sectional | ELISA | 364 | 123 | 8 |
| (Majda Laboudi & Sadak [57] | 2017 | Morocco | Cross-sectional | ELISA | 128 | 59 | 6 |
| Mahdy et al. [58] | 2017 | Yemen | Cross-sectional | ELISA | 359 | 178 | 7 |
| Naghili et al. [59] | 2017 | Iran | Cross-sectional | ELISA | 391 | 267 | 7 |
| Nahouli et al. [60] | 2017 | Lebanon | Cross-sectional | ELISA | 11000 | 2471 | 7 |
| Nazir [61] | 2017 | Pakistan | Cross-sectional | ELISA | 232 | 132 | 8 |
| Norouzi et al. [62] | 2017 | Iran | Cross-sectional | ELISA | 2000 | 173 | 8 |

| | | | | | | | |
|---|---|---|---|---|---|---|---|
| Shieh et al. [63] | 2017 | Iran | Cross-sectional | ELISA | 261 | 127 | 7 |
| Shirdel et al. [64] | 2017 | Iran | Cross-sectional | ELISA | 440 | 189 | 8 |
| Tlamcani et al. [65] | 2017 | Morocco | Cross-sectional | CMIA | 3440 | 1367 | 7 |
| Aleem et al. [66] | 2018 | Pakistan | Cross-sectional | LAT | 360 | 170 | 8 |
| (Eshratkhah Mohammadnejad et al. [67] | 2018 | Iran | Cross-sectional | ELISA | 620 | 117 | 7 |
| (Fallahizadeh et al. [68] | 2018 | Iran | Cross-sectional | ELISA | 276 | 85 | 7 |
| Rashno et al. [69] | 2018 | Iran | Cross-sectional | ELISA | 98 | 34 | 7 |
| Sardarian et al. [70] | 2018 | Iran | Cross-sectional | ELISA | 653 | 167 | 6 |
| Abdul-Ghani et al. [71] | 2019 | Yemen | Cross-sectional | ELISA | 379 | 71 | 6 |
| (Al-Adhroey et al. [72] | 2019 | Yemen | Cross-sectional | ELISA | 420 | 89 | 7 |
| Khademi et al. [73] | 2019 | Iran | Cross-sectional | ELISA | 360 | 103 | 8 |
| Rashno et al. [74] | 2019 | Iran | Cross-sectional | ELISA | 98 | 34 | 8 |
| Mosawi et al. [75] | 2019 | Afghanistan | Cross-sectional | ELISA | 431 | 207 | 7 |
| (Rahmati-Balaghaleh et al. [76] | 2019 | Iran | Cross-sectional | ELISA | 208 | 114 | 7 |
| Sadiqui et al. [77] | 2019 | Pakistan | Cross-sectional | IFFT | 500 | 54 | 6 |
| Sharifi et al. [78] | 2019 | Iran | Cross-sectional | ELISA | 250 | 76 | 7 |
| Abdelbaset et al. [79] | 2020 | Egypt | Cross-sectional | ELISA | 96 | 29 | 7 |
| Al-Hakami et al. [80] | 2020 | Saudi Arabia | Cross-sectional | ELISA | 190 | 52 | 7 |
| Ali et al. [81] | 2020 | Pakistan | Cross-sectional | ELISA | 222 | 30 | 6 |
| Edrees & Ibrahim [82] | 2020 | Iraq | Cross-sectional | ELISA | 150 | 40 | 7 |
| (Hoummadi et al. [83] | 2020 | Morocco | Cross-sectional | ELISA | 5578 | 1607 | 8 |
| (Jahantigh et al. [84] | 2020 | Iran | Cross-sectional | ELISA | 90 | 13 | 8 |

| | | | | | | | |
|---|---|---|---|---|---|---|---|
| Mohajab et al. [85] | 2020 | Saudi Arabia | cross-sectional | ELISA | 2754 | 38 | 8 |
| Qamer et al. [86] | 2020 | Saudi Arabia | Cross-sectional | ELISA | 306 | 102 | 7 |
| Raissi et al. [87] | 2020 | Iran | Case-control | ELISA | 189 | 75 | 8 |
| (Al-Shammari & Iqbal [88] | 2021 | Kuwaiti | Cross-sectional | ELISA | 280 | 41 | 7 |
| Al-Yami et al. [89] | 2021 | Saudi Arabia | Cross-sectional | ELISA | 500 | 105 | 7 |
| (Majda Laboudi et al. [90] | 2021 | Morocco | Cross-sectional | ELISA | 637 | 299 | 8 |
| Mahmood & Kahya [91] | 2021 | Iraq | Case-control | ELISA | 109 | 43 | 7 |
| Soltani et al. [92] | 2021 | Iran | Cross-sectional | ELISA | 88 | 30 | 7 |
| Yusuf et al. [93] | 2021 | Somalia | Cross-sectional | ELISA | 307 | 159 | 7 |
| Nabizadeh et al. [94] | 2022 | Iran | Cross-sectional | CLIA | 2726 | 722 | 8 |
| Hassan et al. [95] | 2023 | Somalia | Cross-sectional | ELISA | 403 | 182 | 7 |

References


1. Kassem HH, Morsy TA. The prevalence of anti-Toxoplasma antibodies among pregnant women in Benghazi, (S.P.L.A.J.) Libya. J Egypt Soc Parasitol. 1991;21:69–74.

2. El-nawawy A, Soliman AT, Azzouni O El, Amer E, Karim MA, Demian S, et al. Maternal and Neonatal Prevalence of Toxoplasma and Cytomegalovirus ( CMV ) Antibodies and Hepatitis-B Antigens in an Egyptian Rural Area. J Trop Pediatr. 1996;42 June:154–7.

3. Dar FK, Alkarmi T, Uduman S, Abdulrazzaq Y, Grundsell H, Hughes P. Gestational and neonatal toxoplasmosis: regional seroprevalence in the United Arab Emirates. Eur J Epidemiol. 1997;13:567–71.

4. Al-mulhim AA, Al-qurashi AM. SERO-PREVALENCE OF TOXOPLASMOSIS IN PREGNANT MOTHERS AND NEW BORN INFANTS IN EASTERN PROVINCE , ولكن ، أعراض أية بلازما التكسو بطفيلي ، كاملة بمناعة يتمتع الذي مائة وخمسة قد تسبب الإصابة بهذا الطفيلي في وقت الحمل مخاطر على الجنين . تسبب إصابة الشخص الطبيعي. J Family Community Med. 2001;8:45–8.

5. Ghazi HO, Telmesani M, Mahomed MF. TORCH Agents in Pregnant. Med Princ Pract. 2002;11:180–2.

6. Noorbakhsh S, Mamishi S, Rimaz S. Toxoplasmosis in Primiparus Pregnant Women and Their Neonates S. Iran J Publ Heal. 2002;31:51–4.

7. Elnahas A, Gerais AS, Elbashir M, Eldien ES, Adam I. Toxoplasmosis in pregnant Sudanese women. Saudi Med J. 2003;24:868-870.

8. Jumaian NF. Seroprevalence and risk factors for Toxoplasma infection in pregnant women in Jordan. East Mediterr Heal J. 2005;11:45–51.

9. Al-harthi SA, Jamjoom M, Hani O. Seroprevalence of Toxoplasma gondii among pregnant women in Makkah , Saudi Arabia Seroprevalence of Toxoplasma Gondii Among Pregnant Women in Makkah , Saudi Arabia باستخدام المكرمة مكة مدينة في لماوحلا النساء عند التوكسوبلازما ليفطب الإصابة نسبة المصلية رات. Umm Al-Qura Univ J Sci Med Eng. 2006;18:217–27.

10. Sharifi-mood B, Hashemi M, Salehi M, Branch S, Naderi M. Seroepidemiology of Toxoplasma Infection in the Pregnant Women in Zahedan , Southeast of Iran. J Res Health Sci. 2006;6 January:2–3.

11. Mansouri B El, Rhajaoui M, Sebti F, Amarir F, Laboudi M, Bchitou R, et al. Seroprevalence of toxoplasmosis in pregnant women in Rabat, Morocco. Bull Soc Pathol Exot. 2007;100:289–90.

12. Iqbal J, Khalid N. Detection of acute Toxoplasma gondii infection in early pregnancy by IgG avidity and PCR analysis. J Med Microbiol. 2007;56:1495–9.

13. Saeedi M, Marjani GRV and A. Seroepidemiologic Evaluation of Anti-toxoplasma Antibodies among Women in North of Iran. Pakistan J Biol Sci. 2007;10:2359–62.

14. Abdi J, Shojaee S, Mirzaee A, Keshavarz H. Seroprevalence of Toxoplasmosis in Pregnant Women in Ilam Province, Iran. Iran J Parasitol. 2008;3:34–7.

15. Fallah M, Rabiee S, And MM, Taherkhani H. Seroepidemiology of toxoplasmosis in primigravida women in Hamadan , Islamic Republic of Iran , 2004. East Mediterr Heal J. 2008;14:163–71.

16. Ibrahim HM, Mohamed AH, El-Sharaawy AA, El-Shqanqery HE. Molecular and serological prevalence of Toxoplasma gondii in pregnant women and sheep in Egypt. Asian Pac J Trop Med. 2017;10:996–1001.

17. Nijem K, Al-Amleh S. Seroprevalence and associated risk factors of toxoplasmosis in pregnant women in Hebron district , Palestine. La Rev Santé la Méditerranée Orient. 2009;15:1278–84.

18. Jameel GH, Al-azzawi AMK, Salman FK. Antigen reaction in detection of toxoplasma gondii antibodies in the serum of pregnant women household pets in Baquba sector. Biochem Cell Arch. 2020;20:3509–11.

19. Mohammad HIA, Amin TT, Balaha MH, Moghannum MSA. Toxoplasmosis among the pregnant women attending a Saudi maternity hospital: seroprevalence and possible risk factors. Ann Trop Med Parasitol. 2010;104:493–504.

20. Sellami H, Amri H, Cheikhrouhou F, Sellami A, Makni F, Trabelsi H, et al. État actuel de la toxoplasmose dans la région de Sfax, Tunisie Toxoplasmosis in Sfax, Tunisia. Bull Soc Pathol Exot. 2010;103:37–40.

21. Almogren A. Antenatal screening for Toxoplasma gondii infection at a tertiary care hospital in Riyadh, Saudi Arabia. Ann Saudi Med. 2011;31 December:569–72.

22. Mohammed TK. SEROPREVALENCE OF TOXOPLASMA GONDII AMONG PREGNANT WOMEN IN BAGHDAD CITY. Inst Med Technol. 2011;23:12–21.

23. Amany MAE-G, and Merwad AMA. Epidemiology and Molecular Detection of Zoonotic Toxoplasma gondii in Cat Feces and Seroprevalence of Anti-Toxoplasma gondii Antibodies in Pregnant Women and Sheep. Life Sci J. 2012;9:133–46.

24. Saad M. Bin Dajem, Almushait MA. Detection of Toxoplasma gondii DNA by PCR in blood samples collected from pregnant Saudi women from the Aseer region, Saudi Arabia. Ann Saudi Med. 2012;32:507–12.

25. El Deeb HK, Salah-Eldin H, Khodeer S, Allah AA. Prevalence of Toxoplasma gondii infection in antenatal population in Menoufia governorate, Egypt. Acta Trop. 2012;124:185–91.

26. Maha H. Elamin. Molecular detection and prevalence of Toxoplasma gondii in pregnant women in Sudan. African J Microbiol Res. 2012;6:308–11.

27. Hajsoleimani F, Ataeian A, Mazloomzadeh S. Seroprevalence of Toxoplasma gondii in Pregnant Women and Bioassay of IgM. Iran J Parasitol. 2012;7:82–6.

28. Babaie J, Amiri S, Mostafavi E, Hassan N, Lotfi P, Rastaghi ARE, et al. Seroprevalence and risk factors for toxoplasma gondii infection among pregnant women in northeast Iran. Clin Vaccine Immunol. 2013;20:1771–3.

29. Ben Abdallah R, Siala E, Bouafsoun A, Maatoug R, Souissi O, Aoun K, et al. Dépistage de la toxoplasmose materno-foetale: étude des cas suivis à l'Institut Pasteur de Tunis (2007-2010). Bull la Soc Pathol Exot. 2013;106:108–12.

30. Fakhfakh N, Kallel K, Ennigro S, Kaouech E, Belhadj S, Chaker E. Facteurs de risque pour toxoplasma gondii et statut immunitaire des femmes parturientes: Relation de cause à effet? Tunisie Medicale. 2013;91:188–90.

31. Panah Abolghasem Siyadat M assadi, Katayoun Bahman Soufiani GB, Gharachorlou A, Zeydi AE. Seroprevalence of Toxoplasma gondii infection among pregnant women in Amol, Northern Iran. Life Sci J. 2013;10:18–21.

32. Abdel-Raouff M, Elbasheir MM. Sero-prevalence of Toxoplasma gondii infection among pregnant women attending antenatal clinics in Khartoum and Omdurman Maternity Hospitals, Sudan. J Coast Life Med. 2014;2:496–9.

33. Ahmed HA, Shafik SM, Alli MEM, Elghamry ST, Ahmed AA. Molecular detection of Toxoplasma gondii DNA in milk and risk factors analysis of seroprevalence in pregnant women at Sharkia, Egypt. Vet World. 2014;7:594–600.

34. Almushait MA, Dajem SMB, Elsherbiny NM, Eskandar MA, Al Azraqi TA, Makhlouf LM. Seroprevalence and risk factors of Toxoplasma gondii infection among pregnant women in south western, Saudi Arabia. J Parasit Dis. 2014;38:4–10.

35. Aqeely H, El-Gayar EK, Perveen Khan D, Najmi A, Alvi A, Bani I, et al. Seroepidemiology of toxoplasma gondii amongst pregnant women in Jazan Province, Saudi Arabia. J Trop Med. 2014;2014.

36. Ashankyty IM. Seroprevalence of Toxoplasma gondii among pregnant women visiting maternity hospital in Hail, KSA. Life Sci J. 2014;11:355–9.

37. Hadi G, Ghomashlooyan M, Hooshyar H. Seroprevalence of Toxoplasma gondii Infection among Pregnant Women Admitted at Shahid Akbar Abadi Hospital, Tehran, Iran, 2010-2013. J Med Microbiol Infec Dis,. 2014;2:16–8.

38. Laboudi M, El Mansouri B, Rhajaoui M. The role of the parity and the age in acquisition of toxoplasmosis among pregnant women in Rabat - Morocco. Int J Innov Appl Stud. 2014;6:488–92.

39. Sharbatkhori M, Moghaddam YD, Pagheh AS, Mohammadi R, Mofidi HH, Shojaee S. Seroprevalence of Toxoplasma gondii infections in pregnant women in Gorgan City, Golestan Province, Northern Iran-2012. Iran J Parasitol. 2014;9:181–7.

40. Yad Yad MJ, Jomehzadeh N, Sameri MJ, Noorshahi N. Seroprevalence of anti-toxoplasma gondii antibodies among pregnant woman in south Khuzestan, Iran. Jundishapur J Microbiol. 2014;7:5–8.

41. Elsafi SH, Al-Mutairi WF, Al-Jubran KM, Abu Hassan MM, Al Zahrani EM. Toxoplasmosis seroprevalence in relation to knowledge and practice among pregnant women in Dhahran, Saudi Arabia. Pathog Glob Health. 2015;109:377–82.

42. Kamal AM, Ahmed AK, Abdellatif MZM, Tawfik M, Hassan EE. Seropositivity of toxoplasmosis in pregnant women by ELISA at Minia university hospital, Egypt. Korean J Parasitol. 2015;53:605–10.

43. Ahmadpour M, Daryani A, Ebrahimnejad Z, Gholami S, Ahmadpour E, Borhani S, et al. Seroprevalence of anti-Toxoplasma IgG and IgM among individuals who were referred to medical laboratories in Mazandaran province, northern Iran. J Infect Public Health. 2016;9:75–80.

44. Akhlaghi L, Tabatabaie F, Hadighi R, Maleki F, Hajialiani F, Dayer MS, et al. Diagnosis of acute toxoplasmosis in pregnant women referred to therapeutic centers of Alborz Province (Iran) using immunoglobulin G avidity ELISA technique. Asian Pacific J Trop Dis. 2016;6:864–7.

45. Al-Eryani SMA, Al-Mekhlafi AM, Al-Shibani LA, Mahdy MMK, Azazy AA. Toxoplasma gondii infection among pregnant women in Yemen: Factors associated with high seroprevalence. J Infect Dev Ctries. 2016;10:667–72.

46. Alghamdi J, Elamin MH, Alhabib S. Prevalence and genotyping of Toxoplasma gondii among Saudi pregnant women in Saudi Arabia. Saudi Pharm J. 2016;24:645–51.

47. Bassiony H, Soliman N, Tawab S, Eissa S, Eissa A. Sero-prevalence and risk factors associated with toxoplasma gondii infection among pregnant women in Alexandria, Egypt. Int J Reprod Contraception, Obstet Gynecol. 2016;5:4220–7.

48. Imam NFA, Azzam EAA, Attia AA. Seroprevalence of Toxoplasma gondii among pregnant women in Almadinah Almunawwarah KSA. J Taibah Univ Med Sci. 2016;11:255–9.

49. Majid A, Khan S, Jan AH, Taib M, Adnan M, Ali I, et al. Chronic toxoplasmosis and possible risk factors associated with pregnant women in Khyber Pakhtunkhwa. Biotechnol Biotechnol Equip. 2016;30:733–6.

50. Mohaghegh MA, Kalani H, Hashemi M, Hashemi S, Yazdnezhad SK, Hejazi SH, et al. Toxoplasmosis-related risk factors in pregnant women in the North Khorasan province, Iran. Int J Med Res Heal Sci. 2016;5:370–4.

51. Mohamed K, Bahathiq A, Degnah N, Basuni S, Mahdi AB, Malki A Al, et al. Detection of Toxoplasma gondii infection and associated risk factors among pregnant women in Makkah Al Mukarramah, Saudi Arabia. Asian Pacific J Trop Dis. 2016;6:113–9.

52. Rostamzadeh Khameneh Z, Hanifian H, Rostamzadeh A. Seroprevalence of Toxoplasmosis in Pregnant Women in Urmia, Iran. Int J Enteric Pathog. 2016;In Press In Press:4–6.

53. Ahmadpour GR, Ezatpour B, Hadighi R, Oormazdi H, Akhlaghi L, Tabatabaei F, et al. Seroepidemiology of Toxoplasma gondii infection in pregnant women in west Iran: determined by ELISA and PCR analysis. J Parasit Dis. 2017;41:237–42.

54. Alanazi FB, Hassan TM, Alanazi WF. Seroprevalence of Toxoplasma gondii among pregnant Saudi woman in Arar, Northern Borders Province, Saudi Arabia. Kasr Al Ainy Med J. 2017;23:104.

55. Garedaghi Y, Firozivand Y. Assessment of pregnant women toxoplasmosis by ELISA method in Miandoab city, Iran. Int J Women's Heal Reprod Sci. 2017;5:72–5.

56. Ibrahim HM, Huang P, Salem TA, Talaat RM, Nasr MI, Xuan X, et al. Prevalence of Neospora caninum and Toxoplasma gondii Antibodies in Pregnant women from Northern Iran. Am J Trop Med Hyg. 2009;80:263–7.

57. Laboudi M, Sadak A. Serodiagnosis of Toxoplasmosis: The effect of measurement of IgG avidity in pregnant women in Rabat in Morocco. Acta Trop. 2017;172:139–42.

58. Mahdy MAK, Alareqi LMQ, Abdul-Ghani R, Al-Eryani SMA, Al-Mikhlafy AA, Al-Mekhlafi AM, et al. A community-based survey of Toxoplasma gondii infection among pregnant women in rural areas of Taiz governorate, Yemen: The risk of waterborne transmission. Infect Dis Poverty. 2017;6:4–9.

59. Naghili B, Abbasalizadeh S, Tabrizi S, Rajaii M, Akramiyan M, Alikhah H, et al. Comparison of IIF, ELISA and IgG avidity tests for the detection of anti-toxoplasma antibodies in single serum sample from pregnant women. Infez Med. 2017;25:50–6.

60. Nahouli H, El Arnaout N, Chalhoub E, Anastadiadis E, El Hajj H. Seroprevalence of Anti-Toxoplasma gondii Antibodies Among Lebanese Pregnant Women. https://home.liebertpub.com/vbz. 2017;17:785–90.

61. Nazir MA. Prevalence of periodontal disease, its association with systemic diseases and prevention. Int J Health Sci (Qassim). 2017;11:72.

62. Norouzi LY, Sarkari B, Asgari Q, Khabisi SA. Molecular Evaluation and Seroprevalence of Toxoplasmosis in Pregnant Women in Fars province, Southern Iran | Annals of Medical and Health Sciences Research. Ann Med Health Sci Res. 2017;7:16–9.

63. Shieh M, Didehdar M, Hajihossein R, Ahmadi F, Eslamirad Z. Toxoplasmosis: Seroprevalence in pregnant women, and serological and molecular screening in neonatal umbilical cord blood. Acta Trop. 2017;174:38–44.

64. Shirdel S, Sharbatkhori M, Pagheh AS, Dadimoghadam Y, Soosaraie M, Gholami S. Seroepidemiology of toxoplasma gondii infection in pregnant women and risk factors of the disease in Golestan Province, Iran. J Maz Univ Med Sci. 2017;27:63–71.

65. Tlamcani Z, Yahyaoui G, Mahmoud M. Prevalence of immunity to toxoplasmosis among pregnant women in UniversityHospital center Hassan II of FEZ city (Morocco). Acta Medica Int. 2017;4:43.

66. Aleem U, Ullah S, Qasim M, Suliman M. Seroprevalence of Toxoplasmosis in Pregnant Women in Matta , Upper Swat , Khyber Pakhtunkhwa , Pakistan. J Saidu Med Coll. 2018;:103–6.

67. Eshratkhah Mohammadnejad A, Eslami G, Shamsi F, Pirnejad A, Samie A, Safabakhsh J, et al. Prevalence of food-borne Toxoplasma in pregnant women population of Urmia, Iran. J Food Qual Hazards Control. 2018;5:17–23.

68. Fallahizadeh S, Jelowdar A, Kazemi F, Cheraghian B. Seroprevalence of Anti-Toxoplasma IgG and IgM among Pregnant Women of Shush County, Southwest of Iran. Int J Infect. 2018;5:1–4.

69. Rashno MM, Fallahi S, Arab-Mazar Z, Dana H. Seromolecular assess of Toxoplasma gondii infection in

pregnant women and neonatal umbilical cord blood. EXCLI J. 2019;18:1.

70. Sardarian K, Maghsood A, Farimani M, Hajiloii M, Saidijam M, Rezaeepoor M, et al. Evaluation of Toxoplasma gondii B1 gene in Placental Tissues of Pregnant Women with Acute Toxoplasmosis. Adv Biomed Res. 2018;7:119.

71. Abdul-Ghani R, Al-Nahari A, Yousef A, Al-Haj A-A, Aqlan A, Jaadan E, et al. Toxoplasma gondii Infection in Relation to Pregnancy Characteristics and Bad Obstetric History among Pregnant Women Seeking Healthcare in Sana'a City, Yemen. Yemeni J Med Sci. 2019;13:10–22.

72. Al-Adhroey AH, Mehrass AAKO, Al-Shammakh AA, Ali AD, Akabat MYM, Al-Mekhlafi HM. Prevalence and predictors of Toxoplasma gondii infection in pregnant women from Dhamar, Yemen. BMC Infect Dis. 2019;19:1–9.

73. Khademi SZ, Ghaffarifar F, Dalimi A, Davoodian P, Abdoli A. Prevalence and risk factors of Toxoplasma gondii infection among pregnant women in Hormozgan province, south of Iran. Iran J Parasitol. 2019;14:167–73.

74. Rashno MM, Fallahi S, Arab-Mazar Z, Dana H. Seromolecular assess of Toxoplasma gondii infection in pregnant women and neonatal umbilical cord blood. EXCLI J. 2019;18:1–7.

75. Mosawi SH, Zarghona Z, Dalimi A. Particularly neglected in countries with other challenges : High Toxoplasma gondii seroprevalence in pregnant women in Kabul , Afghanistan , while a low proportion know about the parasite. PLoS One. 2019;14:1–9.

76. Rahmati-Balaghaleh M, Hosseini Farash BR, Zarean M, Hatami-Pourdehno S, Mirahmadi H, Jarahi L, et al. Diagnosis of acute toxoplasmosis by IgG avidity method in pregnant women referred to health centers in south-eastern Iran. J Parasit Dis. 2019;43:517–21.

77. Sadiqui S, Shah SRH, Almugadam BS, Shakeela Q, Ahmad S. Distribution of Toxoplasma gondii IgM and IgG antibody seropositivity among age groups and gestational periods in pregnant women. F1000Research. 2019;7:1–12.

78. Sharifi K, Hosseini Farash BR, Tara F, Khaledi A, Sharifi K, Shamsian SAA. Diagnosis of acute toxoplasmosis by igg and igm antibodies and igg avidity in pregnant women from Mashhad, eastern Iran. Iran J Parasitol. 2019;14:639–45.

79. Abdelbaset AE, Hamed MI, Abushahba MFN, Rawy MS, Sayed ASM. Toxoplasma gondii seropositivity and the associated risk factors in sheep and pregnant women in El-Minya Governorate , Egypt. Vet World. 2020;13:13–5.

80. Al-Hakami AM, Paul E, Al-Abed F, Alzoani AA, Shati AA, Assiri MI, et al. Prevalence of toxoplasmosis, rubella, cytomegalovirus, and herpes (TORCH) infections among women attending the antenatal care clinic, maternity hospital in Abha, Southwestern Saudi Arabia. Saudi Med J. 2020;41:757–62.

81. Ali S, Amjad Z, Khan TM, Maalik A, Iftikhar A, Khan I, et al. Occurrence of Toxoplasma gondii antibodies and associated risk factors in women in selected districts of Punjab province , Pakistan. Parasitology. 2020;147:1133–1139.

82. Edrees T, Ibrahim RH. Seroprevalence of Toxoplasma Gondii among pregnant women visiting antenatal Clinic at the Mosul City , Iraq . P J M H S. 2020;14:1046–9.

83. Hoummadi L, Berrouch S, Amraouza Y, Adel A, Mriouch M, Soraa N, et al. Seroprevalence of

toxoplasmosis in pregnant women of the marrakech-safi region, morocco. Afr Health Sci. 2020;20:59–63.

84. Jahantigh FF, Rasekh M, Ganjali M, Sarani A. Seroprevalence of Toxoplasma Gondii infection among pregnant women and small ruminant populations in Sistan Region, Iran. Iran J Vet Med. 2020;14:239–50.

85. Mohajab AH, Alshehri HZ, Shati RO, Alshehri AA, Alafghani MA, Alasmari A, et al. Anti-toxoplasma Antibody Prevalence and Cost-effectiveness in Pregnant Women at the King Abdulaziz University Hospital, Jeddah, Saudi Arabia. Cureus. 2020;12:1–8.

86. Qamer S, S.S.R. R, Raoof S, Kamal SM, Khan S. Sero-prevalence of toxoplasmosis among pregnant women attending an ante-natal clinic at a teaching hospital in al Kharj, Saudi Arabia. Trop Biomed. 2020;37:186–93.

87. Raissi V, Taghipour A, Navi Z, Etemadi S, Sohrabi Z, Sohrabi N, et al. Seroprevalence of Toxoplasma gondii and Toxocara spp. infections among pregnant women with and without previous abortions in the west of Iran. J Obstet Gynaecol Res. 2020;46:382–8.

88. Al-Shammari N, Iqbal J. Decreasing trend in toxoplasma seroprevalence among pregnant women in Kuwait. East Mediterr Heal J. 2021;27:67–75.

89. Al-Yami FS, Dar FK, Yousef AI, Al-Qurouni BH, Al-Jamea LH, Rabaan AA, et al. A pilot study on screening for gestational/congenital toxoplasmosis of pregnant women at delivery in the Eastern Province of Saudi Arabia. Saudi Pharm J. 2021;29:343–50.

90. Laboudi M, Taghy Z, Duieb O, Peyron F, Sadak A. Toxoplasma gondii seroprevalence among pregnant women in Rabat, Morocco. Trop Med Health. 2021;49.

91. Mahmood MT, Kahya HFH. Serological study of torch complex in pregnant women with an obstetric history in mosul city, iraq. Curr Trends Immunol. 2021;22:95–102.

92. Soltani S, Ghaffari AD, Kahvaz MS, Sabaghan M, Pashmforosh M, Foroutan M. Detection of Anti-Toxoplasma gondii IgG and IgM Antibodies and Associated Risk Factors during Pregnancy in Southwest Iran. Infect Dis Obstet Gynecol. 2021;2021.

93. Yusuf AA, Hassan-Kadle AA, Ibrahim AM, Hassan-Kadle MA, Yasin AM, Khojaly M, et al. Prevalence of Anti-Toxoplasma gondii and Anti-Brucella Spp. Antibodies in Pregnant Women From Mogadishu, Somalia. Front Reprod Heal. 2021;3 August:1–5.

94. Nabizadeh E, Ghotaslou A, Salahi B, Ghotaslou R. The Screening of Rubella Virus, Cytomegalovirus, Hepatitis B Virus, and Toxoplasma gondii Antibodies in Prepregnancy and Reproductive-Age Women in Tabriz, Iran. Infect Dis Obstet Gynecol. 2022;2022:2–6.

95. Hassan SA, Tamomh AG, Hassan DA, Ahmed NR. Seroprevalence of anti-Toxoplasma gondii antibodies and associated risk factors in pregnant women in Somalia. Acta Trop. 2023;238 September 2022:106774.
